# Impact of periodontitis on circulating cell-free DNA levels as a measure of cardiovascular disease

**DOI:** 10.1007/s00784-023-05300-y

**Published:** 2023-10-10

**Authors:** Gaetano Isola, Alessandro Polizzi, Marco Mascitti, Simona Santonocito, Vincenzo Ronsivalle, Marco Cicciù, Paolo Pesce

**Affiliations:** 1 Department of General Surgery and Surgical-Medical Specialties, Unit of Periodontology, School of Dentistry, University of Catania, Via S. Sofia 78, 95123 Catania, Italy; 2 Department of Clinical Specialistic and Dental Sciences, Marche Polytechnic University, Ancona, Italy; 3 Department of Surgical Sciences and Integrated Diagnostics (DISC), University of Genoa, Genoa, Italy

**Keywords:** Periodontitis, Circulating cfDNA, Cardiovascular disease, Periodontal inflamed surface area, C-reactive protein, Clinical trial, Periodontics

## Abstract

**Objectives:**

The present study aims to assess the serum circulating cell-free (cfDNA) concentrations in patients with periodontitis and cardiovascular disease (CVD) and to evaluate the impact of periodontitis on circulating cfDNA levels and the confounding factors that might mediated the possible relationship.

**Materials and methods:**

Healthy controls (*n*=30) and patients with CVD (*n*=31), periodontitis (*n*=31), and periodontitis + CVD (*n*=30) were enrolled in the present study. All subjects underwent regular periodontal examination and blood sampling and cfDNA evaluation. The analysis of the plasma cfDNA concentrations was performed using a dsDNA Assay Kit.

**Results:**

In comparison with healthy controls and CVD patients, periodontitis and periodontitis+CVD exhibited significantly higher expression of circulating cfDNA (*p*<0.05). There was a positive correlation among plasma cfDNA and clinical attachment loss (CAL) (*p*=0.019), high sensitivity C-reactive protein (hs-CRP) (*p*=0.027), and periodontal inflamed surface area (PISA) (*p*=0.003). Furthermore, the multivariate regression analysis evidenced that PISA (*p*<0.001), hs-CRP (*p*=0.014), and full-mouth bleeding score (FMBS) (*p*=0.004) were significant predictors of circulating cfDNA concentrations.

**Conclusions:**

The results of the study highlighted that the periodontitis and periodontitis + CVD group showed higher circulating cfDNA expression in comparison with healthy controls and CVD patients. Moreover, the extent of periodontitis was correlated with the increased cfDNA levels and represented a significant predictor of the increased circulating cfDNA concentrations.

**Clinical relevance:**

Unbalanced circulating cfDNA concentrations have been indicated to represent a possible risk of CVD and endothelial dysfunction. Periodontitis and periodontitis + CVD patients showed higher circulating cfDNA expression; moreover, the extent of periodontitis significantly predicted higher circulating cfDNA concentrations, suggesting the potential increased risk of developing CVD in periodontitis patients.

**Supplementary Information:**

The online version contains supplementary material available at 10.1007/s00784-023-05300-y.

## Introduction

Periodontitis is a multifactorial inflammatory disease with a bacterial aetiology affecting the periodontium that can generate a persistent inflammatory response which can destroy if not properly and preventively treated, the gingiva, periodontal ligament, connective tissues, and the underlying alveolar bone and that result in tooth loss [1, 2]. According to epidemiological data from the Global Burden of Disease Study 2016, severe forms of periodontitis represent the eleventh most widespread pathological condition worldwide, with a reported prevalence of 20% to 50% of the global population [3]. Furthermore, periodontitis has been reported to be closely associated with various systemic diseases, including obesity [4, 5], metabolic syndrome [6, 7], osteoporosis [8], rheumatic diseases [9], Alzheimer’s disease [10], and cardiovascular diseases (CVDs) [11–13] that could lower the overall quality of life [14].

Specifically, a 2019 workshop co-hosted by the American Academy of Periodontology (AAP) and the European Federation of Periodontology (EFP) concluded that periodontitis represents a significant risk factor for CVDs [15]. It has been shown that the mechanism of how periodontitis can contribute to the genesis and progression of endothelial dysfunction, atherosclerosis, and CVD involves an unbalanced oxidative stress pathway, the release of several systemic pro-inflammatory mediators, the dysregulation of reactive oxygen species (ROS), and the reduction of nitric oxide (NO) availability [16].

In this regard, circulating cell-free DNA (cfDNA) refers to extracellular DNA fragments in the blood and other body fluids [17] and has gained an increased interest as a noninvasive biomarker for disease diagnosis and prognosis of several inflammatory diseases. In a variety of conditions, including CVD [18, 19] and several inflammatory diseases [20], elevated levels of circulating cfDNA have been documented. Furthermore, some preclinical studies have suggested that during the early stages of periodontitis, cfDNA are strictly related to innate immune responses and could be a major inducement for mechanisms involved in periodontal tissue inflammation and alveolar bone loss [21, 22].

Furthermore, endogenous nuclear and mitochondrial cfDNA concentrations have been shown to be released by damaged host cells, and exogenous bacterial or viral DNA serves as ligands for toll-like receptor-9 (TLR9), one of the main important mediators of inflammatory pathways in periodontitis active stages of tissue breakdown [23]. Moreover, the level of cfDNA in gingival crevicular fluid has also been reported to correlate with the severity of periodontitis [24]. In vitro studies also demonstrate that bDNA, as part of cfDNA, can induce periodontitis [25] and, at the same time, relate to some therapeutic target strategies against CVD and endothelial dysfunctions [12, 26]. However, despite these preliminary studies having shown that cfDNA is a potential mediator of periodontitis and CVD, its role in the magnitude of the inflammatory process and as an early risk factor for both periodontitis and CVD is still unclear.

Based on these findings, this study aimed to examine the serum concentration of circulating cfDNA in patients with periodontitis and with CVD. In addition, the secondary outcome was to identify the impact of periodontitis on serum cfDNA expression and to assess the possible confounders that might have influenced this association. The null hypothesis to invalidate was that there were no significant differences among cfDNA levels in the analyzed groups.

## Methods

### Study design

Between November 2019 and June 2022, 321 consecutive subjects were evaluated for eligibility at the Dental School of the University of Catania, Catania, Italy. The research was conducted in accordance with the guidelines for strengthening the communication of observational studies (STROBE) [27] (Supplementary Table 1) and followed the declaration of Helsinki on medical research guidelines reviewed in 2016. The study protocol was registered on ClinicalTrials.gov (NCT05590780), and ethical approval was obtained from the Institutional Review Board of the University of Catania, Catania, Italy (215/21/PO). Before the enrolment stage, each participant signed an informed consent specifying the protocol’s risk and characteristics.

### Study sample

During the initial visit, all subjects underwent an anamnestic examination, which included the documentation of clinical history, pharmacological treatment, and previous clinical records. Periodontitis was identified based on the classification of periodontal diseases [1] with the following inclusion criteria: (1) presence of ≥16 teeth; (2) ≥ 40% of periodontal sites with a probing depth (PD) ≥ 4mm and a clinical attachment level (CAL) ≥ 2mm; (3) bleeding on probing (BOP) in ≥ 40%; (4) alveolar bone loss (ABL) ≥ 2mm in ≥ 2sites, verified through periapical Rinn X-rays.

CVD was diagnosed when percutaneous coronary intervention/coronary angiography revealed 50% stenosis of at least one coronary artery. CVD was diagnosed by the same calibrated operator who analyzed the medical records, and CVD subjects underwent an electrocardiogram to detect the possible presence of atrial fibrillation or other pathologies. The subjects of the periodontitis + CVD study were required to meet the combined inclusion criteria for periodontitis and CVD, whereas individuals were considered healthy controls if they were free of systemic disease and did not take any medications. On periapical Rinn X-rays, neither healthy nor CVD subjects had any periodontal sites with PD and CAL >3 mm, BOP >10%, or ABL >2 mm. Patients with periodontitis and with periodontitis + CVD were classified on the basis of the recent classification of periodontal disease in stages and grades [1].

For all four groups, the following exclusion criteria were adopted: (2) antibiotics, immunosuppressants, anti-inflammatory or any other drugs which could induce gingival hyperplasia in the last 6 months prior to the study; (3) history of alcohol abuse; (4) drug allergies/intolerances; (5) lactation or pregnancy; (6) diabetes or rheumatic diseases; (7) periodontal therapy in the last 6 months prior to the study; (8) COVID positivity status. Healthy patients did not have systemic diseases or mouth disorders and were not subjected to active drug treatment.

**Fig. 1:**
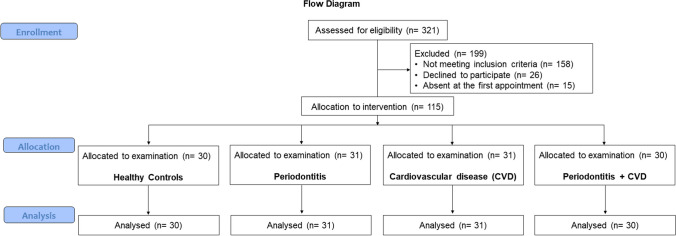
Flowchart of the study

After an initial screening of the 321 screened patients, 199 participants were excluded because they did not meet the study inclusion criteria (*n*= 158), declined to participate (*n*= 26), or were absent at the clinical visit (*n*= 15) (Fig. 1). Finally, 122 subjects were enrolled and divided into four groups: healthy controls (*n*= 30), CVD (*n*= 31), periodontitis (*n*= 31), and CVD + periodontitis (*n*= 30) (Fig. 1).

### Clinical parameters

During the first visit, each selected subject was registered for demographic characteristics such as age, sex, body mass index (BMI), smoking habit (classified as current, never smokers, ex-smokers), presence of comorbidities/medications, and level of education (primary school, college, university). Furthermore, the subjects were classified into three socioeconomic status (SES) levels (low, middle, and high) as a combined measure of education, income and occupation [28]. Glucose levels >125 mg/dL or a relevant medical history were indicative of diabetes. All patients were required to undergo a COVID test at their initial visit beginning in February 2020. Patients who tested positive were excluded from the study.

Regarding periodontal examination, 2 calibrated independent examiners recorded the periodontal indices at six sites per tooth for all present teeth, excluding wisdom teeth, using a standardized periodontal probe^1^. More specifically, PD, CAL, BOP, full-mouth plaque score (FMPS) [29], full-mouth bleeding score (FMBS), and ABL were recorded in each enrolled patient. CAL was assessed by calculating PD and the gingival recession (REC) levels using the cementoenamel junction (CEJ) as a reference, and Periodontal Inflamed Surface Area (PISA) as a measure of both FMPS and CAL was calculated as previously reported [30]. ABL was measured on the distal and mesial alveolar bone levels close to the root surfaces of each tooth using periapical Rinn X-rays.

Inter-and intra-examiner reliability was assessed using PD and CAL as reference values, using the intraclass correlation coefficient (ICC) analysis. There was good agreement among examiners for both PD (ICC= 0.826) and CAL (ICC= 0.823). The intra-examiner reliability for both examiners was performed on only six random subjects per group (24 total subjects). For the first examiner, reliability showed a good level of agreement for both PD (ICC= 0.822) and CAL (ICC = 0.821); for the second examiner (control), reliability was also good for both PD (ICC= 0.817) and CAL (ICC= 0.819).

### Biological samples collection

During the first visit, before the intraoral examination, a single operator took blood samples from each patient between 8:00 and 10:00 a.m. Immediately after collection, blood samples were centrifuged (1000 rpm for 2 min) at 4°C and stored at −80 °C. A nephelometric assay kit was used to measure high-sensitive C-reactive protein (hs-CRP) expressed in milligrams per liter. Serum glucose, triglycerides, total cholesterol, and low- (LDL) and high-density lipoprotein (HDL) cholesterol levels were analyzed through laboratory techniques. Moreover, the arterial blood was collected in 3.2% citrate tubes using K2 EDTA vacutainers and processed within 1 h. Blood treated with EDTA was centrifuged at 2000*g* for 10 min at room temperature to isolate plasma. Until analysis, plasma specimens were stored at −80°C. Due to the potential impact of hemolysis on the concentration of cfDNA, plasma samples were visually examined for hemolysis prior to cfDNA analysis. Hemolysis-detectable plasma samples were excluded from cfDNA analysis. Quantification of the plasma cfDNA was performed using the Quant-iT™ PicoGreen™ dsDNA Assay Kit^2^ following the manufacturer’s instructions. Samples were measured in technical triplicate with 500 μl plasma per well, using a fluorescence microplate reader^3^ at excitation and emission wavelengths of 480 nm and 520 nm, respectively.

### Power sample size analysis

The power sample was determined using statistical software^4^. In agreement with previous studies [24, 31], the sample size was calculated using plasma cfDNA as a primary outcome variable and considering the four analyzed groups of patients. Assuming a power level of 80%, an effect size of 0.34 resulting from a ranged value between 0.25 (medium effect size) and 0.40 (large effect size), and a 2-sided significance level of 0.05, it was fixed a priori that at least 28 patients per group were needed in order to achieve a power level of 80%. However, to avoid potential drop-outs, 122 patients were finally enrolled in order to achieve a power level higher than 85%.

### Statistical analysis

For each of the four groups, numerical data was presented as a median and interquartile range (IQR), whereas categorical variables were presented as absolute frequencies and percentages.

As determined by the Kolmogorov-Smirnov test, the examined variables did not exhibit a normal distribution; consequently, a non-parametric approach was utilized. The Kruskall-Wallis test was used to compare numerical variables between the four groups, while the Dunnet test was used for two-by-two comparisons. Bonferroni’s correction was applied to these multiple comparisons, with the significance alpha level of 0.050 divided by the number of possible comparisons. Consequently, this study’s “adjusted” significance level was 0.050/6= 0.008. The chi-square test was used to compare the four groups’ categorical variables, such as sex, smoking, hypertension, and CVD drugs. Spearman’s correlation test was used to evaluate a possible significant interdependence between plasma cfDNA concentration versus hs-CRP and CAL.

Moreover, univariable and multivariable linear regression models were estimated to assess the dependence of plasma cfDNA concentration from potential variables, including age, sex, BMI, smoking, education, SES, hs-CRP, glycated hemoglobin (HbA1c), fasting glucose, CVD, number of teeth, FMPS, and PISA. Smoking and CVD were included in the model as dichotomous (yes/no) variables. Statistical analyses were performed using statistical software^5^ by a skilled statistician blinded to the study groups. A *p*-value lower than 0.05 was considered statistically significant.

## Results

The clinical characteristics of the sample are represented in Table 1. All participants were 40 to 65 years old with a 1:1 female/male ratio to avoid any difference and were well matched for age (*p*= 0.547), gender (*p*= 0.236), number of smokers, and education.

**Table 1:** Patients clinical characteristics. Values are indicated as: 50° percentile (25°–75° percentiles) or number and percentage

Characteristics	Controls (*n*= 30)	CVD (*n*= 31)	Periodontitis (*n*= 31)	Perio+CVD (*n*= 30)	*p*-value*
Age	49.6 (47.1–53.5)	51.4 (50.1–54.2)	52.1 (50.5–56.0)	52.9 (50.2–54.3)	0.547
Gender (male/female)	16/14	15/16	14/17	14/16	0.236
*Education. n* (%)					
Primary school. *n* (%)	9 (30)	10 (32.3)	11 (35.4)	9 (30)	0.148
College. *n* (%)	13 (43.3)	11 (35.4)	11 (35.4)	12 (40)	0.258
University. *n* (%)	8 (26.7)	10 (32.3)	9 (29.1)	9 (30)	0.334
*Smokers. n* (%)					
Never smokers. *n* (%)	29 (96.7)	30 (96.7)	29 (93.6)	28 (93.3)	0.441
Current smokers. *n* (%)	1 (3.3)	1 (3.3)	2 (6.4)	2 (6.7)	0.236
Periodontitis stages (EFP/AAP)					
Stage III	–	–	21 (67.7)	22 (73.3)	0.232
Stage IV	–	–	10 (32.3)	8 (26.7)	0.445
Periodontitis grade (EFP/AAP)					
Grade B	–	–	18 (58)	19 (63.3)	0.326
Grade C	–	–	13 (42)	11 (36.7)	0.595
hs-CRP	3.54 (2.10–4.12)	6.03 (5.43–6.78)	6.39 (5.89–7.11)	6.78 (6.31–7.66)	<0.001
BMI	24.22 (21.59–26.45)	25.11 (22.23–25.85)	25.64 (22.15–26.29)	26.21 (23.66–27.74)	0.317
N° tooth	25.00 (24.00–28.00)	23.00 (22.00–25.90)	20.50 (19.00–24.45)	21.40 (19.85–22.40)	<0.001
HbA1c	5.12 (4.66–5.33)	5.28 (4.89–5.48)	5.45 (5.02–5.36)	5.51 (5.27–5.69)	0.089
Fasting glucose	114.00 (95.00–123.00)	119.00 (102.00–127.00)	121.80 (112.50–128.00)	120.10 (110.00–128.50)	0.188
cfDNA	1235.4 (1194.3–1356.3)	1278.7 (1195.5–1336.5)	1386.2 (1289.6–1421.5)	1409.3 (1362.5–1496.3)	0.039
PD	2.19 (1.62–2.27)	2.36 (2.23–2.88)	4.56 (4.16–4.82)	4.41 (4.12–4.87)	<0.001
PISA	202.40 (117.75–253.20)	301.20 (122.40–312.40)	1710.15 (1639.40–1886.40)	1889.40 (1466.23–2327.40)	<0.001
CAL	2.12 (1.82–2.36)	2.32 (2.13–2.58)	3.98 (3.82–4.39)	4.87 (3.66–4.96)	<0.001
FMPS	8.12 (6.12–9.85)	12.41 (6.46–15.59)	51.42 (46.85–55.58)	53.45 (47.85–56.74)	<0.001
FMBS	7.45 (5.79–8.85)	8.59 (7.45–9.95)	47.48 (43.45–49.45)	48.79 (45.66–49.45)	<0.001

The results of the Dunnet Test revealed that, in comparison with healthy controls, the CVD group showed higher hs-CRP levels (*p*<0.01) (Table 2). However, in comparison with CVD, patients with periodontitis (*p*<0.001) and periodontitis+CVD (*p*= 0.048, respectively) showed higher hs-CRP levels, as well as PD, CAL, FMBS, FMPS, PISA, and a lower number of teeth (*p*<0.05 for all comparisons) (Tables 1 and 2).

**Table 2:** Pairwire comparisons among variables obtained through the Dunnet test. Ctrl, healthy controls; *CVD* cardiovascular disease

Variable	Ctrl vs Perio	Ctrl vs CVD	Ctrl vs Perio+CVD	Perio vs CVD	Perio vs Perio+CVD	CVD vs Perio+CVD
CRP	<0.001	<0.001	<0.001	<0.001	0.441	0.048
N° tooth	<0.001	0.061	<0.001	<0.001	0.378	<0.001
HbA1c	<0.001	0.066	0.059	0.123	0.881	0.086
Fasting glucose	<0.001	0.057	0.109	0.133	0.205	0.493
PD	<0.001	0.074	<0.001	<0.001	0.397	<0.001
CAL	<0.001	0.079	<0.001	<0.001	0.415	<0.001
FMBS	<0.001	0.136	<0.001	<0.001	0.657	<0.001
PISA	<0.001	0.071	<0.001	<0.001	0.184	0.022
FMPS	<0.001	0.057	<0.001	<0.001	0.432	<0.001
Plasma fDNA	0.002	0.049	0.006	0.048	0.552	0.046

### cfDNA concentration

**Fig. 2:**
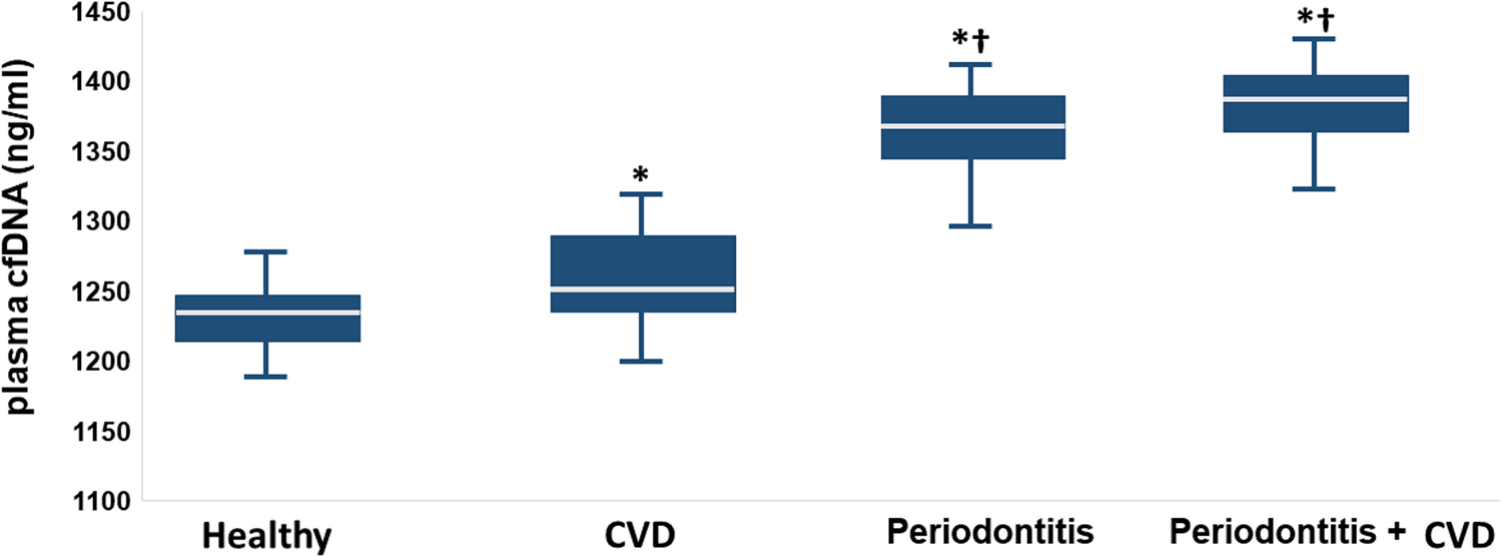
Mean values of plasma cfDNA concentrations (ng/ml). *p < 0.05 significant differences vs healthy controls; ^†^*p* < 0.05 significant differences vs CVD patients

**Fig. 3:**
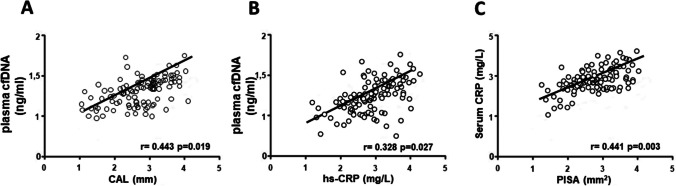
Correlation analysis among plasma cfDNA concentrations and **A** CAL, **B** hs-CRP, and **C** PISA

Regarding plasma cfDNA concentration among groups, the pairwise comparison highlighted that, compared to healthy controls, CVD patients showed higher plasma cfDNA levels (*p*= 0.049) (Table 2, Fig. 2). However, in comparison with healthy controls (*p*= 0.002) and CVD group (*p*= 0.048), the periodontitis group showed higher plasma cfDNA levels. Moreover, compared to CVD, the periodontitis+CVD group presented significantly higher plasma cfDNA levels (*p*= 0.046). At the same time, there were no differences among patients with periodontitis and periodontitis + CVD (*p*= 0.552) (Table 2, Fig. 2). Furthermore, the correlation analysis evidenced that, in all patients, there was a positive correlation among plasma cfDNA and CAL (rs= 0.443, *p*=0.019), hs-CRP (rs= 0.328, *p*= 0.027), and PISA (rs= 0.441, *p*= 0.003), while the other variables analyzed were insignificant (Fig. 3).

### Uni- and multivariate analysis

The uni- and multivariable regression models were estimated to identify significant predictors of plasma cfDNA concentration (Table 3). In the univariate model, HbA1c (*p*=0.015), FMPS *(p*=0.004), and PISA (*p*<0.001) were significant predictors of plasma cfDNA concentration. The multivariate model analysis, in which only significant variables in the univariate model were inserted, evidenced that PISA (*p*<0.001), FMBS (*p*=0.004), and hs-CRP (*p*=0.014) were significant predictors of plasma cfDNA concentration. The other analyzed variables were not significant (Table 3).

**Table 3:** Uni- and multivariable linear regression analysis for plasma cfDNA concentrations in all enrolled patients. Significant values *p*<0.05

Variables	Plasma circulating cfDNA			
	Univariate model		Multivariate model	
	*B*	*p*-value	*B*	*p*-value
Age	0.632	0.633	–	–
Gender	0.985	0.436	–	–
BMI	0.058	0.087	–	–
Smoking	0.839	0.128	–	–
Education	0.552	0.369	–	–
SES	0.441	0.548	–	–
hs-CRP	0.671	0.046	0.318	0.014
HbA1c	0.484	0.122	–	–
Fasting glucose	0.678	0.219	–	–
CVD	0.368	0.196	–	–
N° Tooth	0.932	0.419	–	–
PISA	0.389	0.021	0.270	<0.001
FMBS	0.985	0.044	0.225	0.004
FMPS	0.158	0.112	–	–

## Discussion

The aim of this study was to assess plasma cfDNA concentration in healthy individuals and subjects with periodontitis and CVD and to determine the impact of periodontitis and CVD on plasma cfDNA concentration in relation to possible CVD risk development.

The results evidenced that cfDNA were significantly different among the 4 groups of patients (*p*<0.001). In this regard, all analyzed groups were similar in age, gender, and the number of smokers. In contrast to the results of the present study, previous evidence reported that current smoking habits might lead to unbalanced expression of plasma cfDNA concentration [32, 33]. This may be due to the fact that smoking could influence cfDNA levels through specific pathways involving several inflammatory mediators such as interleukin (IL)-6, IL-8, and proteins p53 [32, 33].

The pairwise analysis among groups evidenced that, compared to healthy controls, the CVD patients had higher cfDNA serum levels. In agreement, it has been previously shown that endothelial dysfunction and atherosclerosis during CVD have been related to upregulated cfDNA concentration [34], although a rise in cardiac cfDNA concentration circulating myeloperoxidase-DNA complexes, a marker for neutrophil extracellular traps (NETs) release that was reported to an early biomarker in patients predisposed to CVD risk [35]. The increased cfDNA concentrations have also been linked with an unbalanced mobilization of endothelial progenitor cells that could stimulate a concomitant relative risk of endothelial dysfunction in patients with CVD [36]. In several inflammatory diseases, including sepsis, pulmonary inflammation, thrombocytopenia, and endothelial dysfunction status, excessive production of cfDNA, nucleosomes, and histones has been demonstrated to be detrimental [37] through a complex network of cellular and molecular interactions which bridge innate and adaptive immunity in atherogenesis and oxidative stress, with subsequent citrullination and a NET formation, which finally could further determine the increased risk of CVD [38]. Moreover, the NET system, in the gingival sulcus of a healthy patient, typically allows the removal of bacterial, pathogen-associated molecular patterns (PAMPs), and damage-associated molecular patterns (DAMPs) that physiologically avoids bacteria colonization on the gingival cells [39]. During periodontitis, the chronic stimulus determined by the load of subgingival pathogens increases the level of the NET system, causing the alteration of homeostasis and the upregulation of cfDNA and released peptides, which ultimately determines chronic gingival inflammation, and that may represent an inflammatory stimulus chronic negative subclinical for the development of systemic diseases [40, 41]. In this regard, a strong relationship was observed between NETs and the release of TRL9 against periodontal pathogens bacteria [42], in which cfDNA has been demonstrated to have a critical role in the interaction between TRL9 and several inflammatory mediators (e.g., ILs, metalloproteinases) that are active during the early stages of periodontitis and in alveolar bone inflammation [31, 43]. Furthermore, cfDNA has also been related to an unbalanced pathway linked with an upregulation of CRP, soluble urokinase-type plasminogen activator receptor (suPAR), and a reduction of NO availability, which may represent a real early risk factor for angiogenic progenitor cell dysfunction and future CVD events during periodontitis [44, 45].

In agreement, the present study results showed that, in comparison with the CVD group, both patients with periodontitis and periodontitis + CVD presented higher plasma cfDNA and hs-CRP concentrations. In this regard, some studies reported that a significant increase of cfDNA mutations was found in patients with forms of periodontal disease; this mechanism was demonstrated to elicit a precise immune response with the involvement of microbial/cytosolic nucleic acids to induce potent immune responses and the innate release of mediators, including type I interferon, which finally regulates the mechanism of gingival and alveolar bone tissue destruction [31, 46].

Moreover, the multivariate linear regression analysis of the present study evidenced that hs-CRP and the extent of periodontitis (PISA and FMBS) were significant predictors of plasma cfDNA concentrations. In agreement, it has been reported that periodontal bacteria could influence the activities of DNases through specific common pathways between the most common bacteria associated with advanced periodontitis and its extent. A study by Zhu et al. [47] reported that upregulated plasma cfDNA concentrations in patients with periodontitis were positively correlated with the extent of periodontal indices. Furthermore, the local increased levels of cfDNA, serving as the ligand to TRL9, also modulates the collection of endogenous DNA released by damaged host cells and exogenous bacterial or viral DNA, a critical pathway associated with the modulation of the innate immunity during periodontitis [25, 48]. Through further and concomitant secretion of CRP, NO, and several related inflammatory mediators [49, 50], this mechanism could determine an additional negative stimulus to further develop CVD and related endothelial dysfunction in predisposed subjects [49, 50].

However, the present study has some limitations that should be addressed, including the study design and the number of patients to be analyzed. A prospective design, together with a more significant number of patients enrolled, could have more specifically determined the effect of cfDNA concentrations as a risk factor in patients with periodontitis. Furthermore, it would be helpful to study the impact of cfDNA levels by evaluating their association with other serum mediators of CVD risk and coagulation mediators and related risks.

## Conclusion

During the last few decades, several studies have tried to find even more valuable biomarkers for the early and subclinical diagnosis of systemic inflammatory disease risk, such as CVD in periodontitis patients. The present study showed that, compared to healthy subjects and CVD, subjects with periodontitis and with periodontitis+CVD had upregulated serum cfDNA concentrations. Furthermore, periodontitis and its severity have significantly predicted serum cfDNA concentrations and the relative subclinical risk of CVD in periodontitis patients. The results of the present preliminary study are promising and open future scenarios on both the diagnostic and therapeutic target of serum cfDNA as a potential biomarker of CVD risk in patients with periodontitis. However, further studies are needed to understand better the impact of periodontitis on serum cfDNA concentrations.

### Supplementary information

ESM 1

## Data Availability

Data are available from the corresponding author upon reasonable request.

## Footnotes

1 UNC-15, Hu-Friedy, Chicago, IL, USA

2 Molecular Probes, Invitrogen

3 BioTek Epoch

4 G* Power version 3.1.9.4, Universitat Dusseldorf, Germany

5 SPSS 22.0 for Windows package, SPS srl, Bologna, Italy
